# Age–Stage, Two-Sex Life Table Analyses of *Zeugodacus tau* Preferences Comparing *Trichosanthes kirilowii* and *Cucurbita moschata*

**DOI:** 10.3390/insects17050462

**Published:** 2026-04-30

**Authors:** Yu-Qi Peng, Jing-Qi Liu, Yan-Yan Xu, Jing-Yu Li, Hai-Gang Liu, Wen-Xiu Guo, Sha Liu, Yi Yu

**Affiliations:** 1 Key Laboratory of Green Prevention and Control of Agricultural Pests in Shandong Province, Plant Protection Research Institute of Shandong Academy of Agricultural Sciences, Jinan 250100, China; 2 State Key Laboratory of Green Pesticide, China–Sri Lanka Belt and Road Joint Laboratory on Tea Ecological Control, Center for R&D of Fine Chemicals of Guizhou University, Guiyang 550025, China; 3 College of Agricultural Science and Technology, Shandong Agriculture and Engineering University, Jinan 250100, China; 4 College of Agronomy and Horticulture Technology, Yunnan Vocational College of Agriculture, Kunming 650212, China

**Keywords:** *Zeugodacus tau*, *Trichosanthes kirilowii*, age–stage, two-sex life table, host suitability, fecundity, population projection

## Abstract

*Zeugodacus tau* Walker (Diptera: Tephritidae) is a quarantine pest of cucurbit crops in China. In recent years, the damage it has caused to *Trichosanthes kirilowii* (Cucurbitales: Cucurbitaceae) has increased, but evidence for host adaptation and population growth on this plant remains limited. We investigated the suitability of *T. kirilowii* as a host plant for *Z. tau*, compared with the known suitable host *Cucurbita moschata*, by examining adult olfactory preference, oviposition choice, and demographic performance using an age–stage, two-sex life table. Females preferred *T. kirilowii* significantly more than *C. moschata* in cases of both peeled and unpeeled fruits, whereas males showed no significant difference. Oviposition was also higher on *T. kirilowii* and further increased on peeled fruit. Developmental duration, total longevity, ovipositing days, oviposition period, and hatching rate did not differ between the two host species. However, *Z. tau* reared on *T. kirilowii* exhibited significantly shorter APOP and TPOP, along with higher fecundity and increased pupal weight. Life table parameters indicated higher intrinsic (*r* = 0.1028 ± 0.0040 d^−1^) and finite rates of increase (λ = 1.1083 ± 0.0044 d^−1^) on *T. kirilowii*, whereas *R*_0_ was similar and mean generation time was shortened by 11.61 d. A 100-day projection predicted 11,980 adults from *T. kirilowii* compared to 1231 from *C. moschata.* Overall, *T. kirilowii* is a highly suitable host that supports the rapid population growth of *Z. tau.*

## Simple Summary

*Zeugodacus tau* is a quarantine pest of cucurbit crops in China; in recent years, it has increasingly affected *Trichosanthes kirilowii*, but evidence on how this plant species can act as a suitable host is limited. In this study, we investigated the host suitability of *T. kirilowii*, compared with the known suitable host *Cucurbita moschata*, by testing the host preference and survival, development, and egg production of *Z. tau* throughout its life stages. Adults were more strongly attracted to and laid more eggs on *T. kirilowii*. *Zeugodacus tau* reared on *T. kirilowii* produced heavier pupae and completed development a generation sooner than those on *C. moschata*, indicating faster population build-up. Population projections further showed that *Z. tau* numbers could increase much more rapidly on *T. kirilowii* within the same time period. These findings suggest that *T. kirilowii* is a highly suitable host for *Z. tau*.

## 1. Introduction

*Zeugodacus tau* Walker (Diptera: Tephritidae) is a serious insect pest of cucurbit crops across Asia. The number of generations of *Z. tau* varies significantly across different regions (typically from one to multiple generations per year), with substantial generational overlap commonly observed [1,2]. *Z. tau* shows broad preferences and adaptability to multiple cucurbit host plant species [3]. Females typically lay eggs inside fruits, where the larvae feed on the pulp and pupate within the fruit, resulting in fruit deformation, premature abscission, or rot, severely reducing both fruit quality and yield. Several studies indicate that cucurbit plant species are favorable for both *Z. tau* adult oviposition and larval development [4,5].

Notably, insects often exhibit distinct survival and reproductive strategies across different host plants, and in tephritid fruit flies, these differences are mainly reflected in the combined effects of host fruits on oviposition choice and offspring development. This pattern has been widely demonstrated across multiple fruit fly species. For example, *Z. cucurbitae* (Coquillett) showed the highest numbers of flower visits and oviposition punctures on *Cucumis sativus* L. and *Cucurbita pepo* L. [6]. These differences in preference are also closely associated with host fruit characteristics [7] such as skin, maturity, and nutritional status, which can influence adult feeding and oviposition behavior and further determine egg–larval survival and development. Theron et al. reported that *Z. dorsalis* shows significantly higher oviposition preference for damaged citrus fruits [8], while Papadopoulos et al. reported that *B. correcta* had a strong oviposition preference for cucurbit fruits, especially when ripe, leading to enhanced larval survival [9]. Overall, host nutritional composition, secondary metabolites, and volatile organic compounds jointly influence adult host location and oviposition behavior, while the physical traits of fruits further constrain oviposition feasibility and larval developmental suitability. Females therefore regulate offspring survival, development, and reproductive performance through host selection, optimizing fitness and influencing population establishment and expansion. Previous studies have shown that *Cucurbita moschata* is a highly suitable host for *Z. tau* [2]. *Trichosanthes kirilowii* (Cucurbitales: Cucurbitaceae) is native to China and East Asia and holds importance in this area due to both its medicinal and culinary uses [10,11]. It is rich in moisture and nutrients [12], which may be favorable for *Z. tau* feeding, oviposition, and larval development. Due to the rapid expansion of *T. kirilowii* production acreage in China, the population density of *Z. tau* and the severity of damage it causes in *T. kirilowii* fields have shown a simultaneous increasing trend, causing significant economic losses [13]. However, evidence regarding the host adaptation of *Z. tau* to *T. kirilowii* remains limited.

Biological characteristics are fundamental attributes of pest species and can serve as an essential basis for establishing IPM systems [14,15]; therefore, a thorough understanding of *Z. tau*’s basic attributes is necessary. Life tables are fundamental tools in the study of population ecology [16], used to determine the survival and reproductive potential of insect populations in certain host plant species and environmental conditions. As outlined by Chi [17], the age–stage, two-sex life table improves upon traditional life tables by incorporating both sexes and life stages [18]. This approach can accurately reflect the life history of insect species and has been widely applied to ecological studies of insect pests and their natural enemies, and pest population dynamics are influenced by life-history traits, habitat conditions, and environmental changes. [19]. Understanding the population parameters derived from age–stage, two-sex life tables is essential in order to conduct accurate risk assessments and establish management strategies in *T. kirilowii* production systems.

In this study, we evaluated the population performance of *Z. tau* on *T. kirilowii* in comparison with a known suitable host *C. moschata*. Population parameters, including developmental duration, survival, and fecundity, were quantified using age–stage, two-sex life tables. In addition, we also compared adult olfactory and oviposition preferences between the two host species. By doing so, we aimed to investigate the adaptation of *Z. tau* to *T. kirilowii* and clarify host-dependent differences in population performance and behavior, thereby providing a basis for pest management in *T. kirilowii* production systems.

## 2. Materials and Methods

### 2.1. Insect Rearing

An experimental colony of *Z. tau* was obtained from the insectary of the Institute of Plant Protection, Shandong Academy of Agricultural Sciences, Jinan, China, where the population has been continuously maintained under laboratory conditions for eight generations. Adult *Z. tau* were reared in insect cages (30 × 30 × 30 cm) and provided with fresh *C. moschata* as food and an oviposition substrate. After oviposition, fruits containing eggs were transferred into slanted glass rearing bottles (2200 mL) and maintained in an illuminated incubator at 25 ± 1 °C, 55–65% relative humidity, and a photoperiod of 16L:8D until larvae developed into mature stages and exited the fruit. Prior to the experiment, late third-instar larvae were selected and placed into sterilized disposable 300 mL plastic containers. Each container was filled with a layer of fine sand approximately 3 cm thick with a gravimetric moisture content of 10~12% to serve as a pupation substrate. There were 20 0.1 mm diameter holes in the container lid.

After pupation, pupae were monitored and recorded until adult emergence. During adult rearing, fresh pieces of *T. kirilowii* and *C. moschata* fruit (approximately 5 cm × 5 cm × 5 cm per piece) were provided daily. After adults mated, the eggs laid were collected and used for subsequent experiments. All eggs, larvae, pupae, and adults were reared in a GXZ380B insect growth chamber (Ningbo Jiangnan Instrument Factory, Ningbo, China) under controlled conditions of 25 ± 1 °C, 55–65% relative humidity, and a photoperiod of 16:8 h (L:D).

### 2.2. Host Plants

*T. kirilowii* and *C. moschata* host fruits used in the experiments were purchased from a local supermarket in Jinan, Shandong Province, China. The selected fruits were fresh with a uniform maturity and free from visible disease symptoms, insect damage, or mechanical injury. All fruits in the market complied with local regulations on pesticide residue monitoring and management. To further reduce potential effects of pesticide residues on the experiment, all host fruits were thoroughly rinsed with tap water, subsequently soaked in sterile water for 2 h, and air-dried at room temperature before use.

### 2.3. Z. tau Olfactory Preference Assay

The experiment was conducted based on the method described by Cornelius, Duan, and Messing [20], with slight modifications. In each insect rearing cage (40 cm × 40 cm × 40 cm), 10-day-old *Z. tau* adults (20 females and 20 males) were released. Fresh, fully mature fruits of *T. kirilowii* and *C. moschata* were used as host materials. For each host, two treatments were set up (peeled vs. unpeeled) and randomly arranged within the cage. For the unpeeled treatment, only the peel side was exposed upward, while all other sides were tightly wrapped with plastic film to minimize interference from non-target surfaces; for the peeled treatment, the peel was removed and the material was prepared in the same manner. Each host was cut into cubes (3 cm × 3 cm × 3 cm), and one cube per host was placed in each cage and randomly arranged at equal distances. Subsequently, the rearing cage was gently tapped for 1 min to induce flight activity in all adults. They were then allowed to settle, and the number of landings shown by male and female adults on each host cube was recorded within 2 min after settling. The experiment was repeated 10 times.

### 2.4. Z. tau Oviposition Preference Assay

This study included three independent choice-preference assays, each conducted in a separate insect rearing cage (30 cm × 30 cm × 30 cm). (1) Unpeeled group: two host fruits (*T. kirilowii* and *C. moschata*) were tested under unpeeled conditions. (2) Peeled group: the same hosts were tested after peeling. (3) Effect-comparison group: To distinguish this group from the aforementioned experiments, an additional independent experiment was conducted. Using *T. kirilowii* as a model host, only two treatments (peeled vs. unpeeled) were set up to specifically evaluate the effect of peeling on the oviposition preference of *Z. tau*. This experiment was designed as a single-factor comparison, and the resulting data were not combined with those from the previous experiments.

Host preparation followed the methods of Zhang et al. [21]. Healthy, intact fruits were selected and completely wrapped in plastic film, and a square window (2 cm × 2 cm) was cut on the film surface (without damaging the peel). The cutting knife was deodorized after each use to avoid interference from residual odors. In each cage, 20-day-old adult *Z*. *tau* were released (10 females and 10 males). After 24 h exposure, the fruits were examined, and the number of eggs deposited within the window area on each host was counted under a stereomicroscope. Each treatment was replicated five times.

### 2.5. Effects of Host Plants on the Development and Population Parameters of Z. tau

Under the experimental condition, an age–stage, two-sex life table of *Z. tau* was constructed using *T. kirilowii* and *C. moschata* as tested hosts. Fresh host materials were placed in separate rearing containers to maintain adults. After mating and oviposition, the most recent 150 eggs laid on each host were collected within 12 h. During the egg stage, observations were conducted twice daily (09:00 and 21:00) to record egg developmental duration and hatching rate.

After hatching, neonate larvae from each host treatment were reared individually [22] in disposable 25 mL transparent plastic cups lined with filter paper, with one larva per cup. Throughout the experiment, fresh pieces of the corresponding host (2 cm × 2 cm × 2 cm) were provided daily to ensure sufficient and fresh food. Larval development and survival were checked and recorded daily. Molting or changes in morphological characteristics were used as indicators of instar transitions, and exuviae were removed promptly after molting.

When mature larvae appeared, they were transferred to 30 mL cups containing sterilized moist sand (20% moisture content) and covered with a 1 cm layer of sterile soil for pupation. After 3 days of pupation, pupae were collected and any adhering sand or surface moisture was removed. They were weighed using an electronic balance (Intelligent LF-225 DR Semi-Micro Balance (Shinko Denshi Co., Ltd., Tokyo, Japan)). After weighing, pupae were returned to the plastic cups and covered with approximately 1 cm of sand until adult emergence. Newly emerged adults were collected every 24 h; then, the emergence time was recorded, sex was determined, and the female-to-male ratio was calculated [22].

Newly emerged males and females were paired at a ratio of 1:1. Adults from the same host treatment were allowed to mate and oviposit in semi-transparent plastic containers (height 4.3 cm; top diameter 11.6 cm; bottom diameter 9.4 cm). The corresponding host material and a diet solution were provided continuously in each container. The diet solution was prepared by mixing sucrose, enzymatic hydrolyzed casein, and water at a ratio of 9:3:100, and it was supplied on absorbent cotton. Host materials and diet solution were replaced daily. From adult emergence onward, the actual ovipositing days and cumulative fecundity (*F*) of each female were recorded daily, and the durations of the adult pre-oviposition period (APOP), total pre-oviposition period (TPOP), and oviposition period (OP) were calculated [23]. Eggs laid by females were collected and incubated concurrently to determine the hatching rate. Observations continued until all adults died naturally. Female longevity, male longevity, and full life-cycle data were obtained for subsequent life table construction and population parameter estimation.

### 2.6. Life Table Data Analysis

Life table data were analyzed using TWOSEX-MSChart (Ver. 2/5/2025) [24] based on the age–stage, two-sex life table theory to evaluate the life table of *Z. tau*. The key parameters calculated were the age–stage-specific survival rate (*S_xj_*), age-specific survival rate (*l_x_*), female age-specific fecundity (*m_x_*), age-specific net maternity (*l_x_m_x_*), intrinsic rate of increase (*r*), finite rate of increase (*λ*), net reproductive rate (*R*_0_), and mean generation time (*T*), following the procedures described by Chi and Liu [25]. The values of *r* and *λ* were reported to four decimal places to ensure numerical precision and comparability of the results [26]. The calculation methods for each parameter were as follows:

*m* denotes the number of life stages, *x* denotes age, and *j* denotes the stage (instar). *S_xj_* represents the probability that a newly laid egg survives to age *x* and stage *j* [27]. The age-specific survival rate is calculated as follows [28]:

(1) $$l_{x}=\sum_{j=1}^{m}S_{xj}$$

*E_i_* denotes the total number of eggs laid by the *x*th female adult, and *N_f_* represents the total number of females. The mean female fecundity (*F*) is calculated as follows [29]:

(2) $$F=\frac{\sum_{x=1}^{N_{f}}E_{i}}{N_{f}}$$

The female age-specific fecundity (*m_x_*) was calculated as follows [30]:

(3) $$m_{x}=\left(\sum_{j=1}^{K}S_{xj}f_{xj}\right)÷\sum_{j}^{k}S_{xj}$$

Using the *Euler–Lotka* equation and the iterative bisection method, the intrinsic rate of increase (*r*) was determined as follows [28]:

(4) $$\sum_{x=0}^{\infty }e^{-r\left(x+1\right)}l_{x}m_{x}=1$$

The finite rate of increase (*λ*) was calculated as follows [31]:

(5) $$\lambda =e^{r}$$

The net reproductive rate (*R*_0_) was calculated as follows [32]:

(6) $$R_{0}=\sum_{x=0}^{\infty }l_{x}m_{x}$$

The mean generation time (*T*) was calculated as follows [27,33]:

(7) $$T=\frac{\ln R_{0}}{r}$$

The age–stage life expectancy (*e_xj_*) represents the expected remaining lifespan of an individual at age *x* and stage *j* [17]. Here, *e_xj_* is calculated based on the probability that an individual at age *x* and stage *j* survives to age *i* and stage *y*, under the assumption that *S′_iy_* = 1. The life expectancy is calculated as follows [27]:

(8) $$e_{xj}=\sum_{i=x}^{\infty }\sum_{y=j}^{m}{S^{\prime }}_{iy}$$

The age–stage reproductive value (*v_xj_*) represents the expected contribution of an individual at age *x* and stage *j* to future population growth, as defined by Fisher [34,35]. The reproductive value is calculated as follows:

(9) $$V_{xj}=\frac{e^{r\left(x+1\right)}}{S_{xj}}\sum_{y=j}^{m}S_{iy}^{\prime }f_{iy}$$

### 2.7. Data Analysis

For the odor and oviposition choice assays, the selections made by *Z. tau* between different host types were analyzed using a *t*-test in IBM SPSS Statistics 25 software, with the significance level set at *p* < 0.05 [36], as were the pupal weight and hatching rate. All *Z. tau* life table data were analyzed using the TWOSEX-MSChart program (Ver. 2/5/2025) [34]. Because the bootstrap procedure involves random resampling, minimizing estimation error is critical. To estimate variances and standard errors, paired bootstrap tests were performed with 100,000 resamplings [27]. Population dynamics were projected using the TIMING-MSChart program (Ver. 18/2/2025) [37,38]. OriginPro 2021 software was used to plot survival rate, fecundity, life expectancy, reproductive value, and population projection curves [39].

## 3. Results

### 3.1. Olfactory Preferences of Z. tau

Table 1 shows that, under both the unpeeled and peeled conditions, female *Z. tau* landed significantly more frequently on *T. kirilowii* than on *C. moschata* (*p* = 0.001; *p* = 0.025). In contrast, no significant differences were observed in the landing frequencies of male *Z. tau* between *T. kirilowii* and *C. moschata* under either condition (*p* = 0.433; *p* = 0.342).

### 3.2. Oviposition Preferences of Z. tau

**Figure 1:**
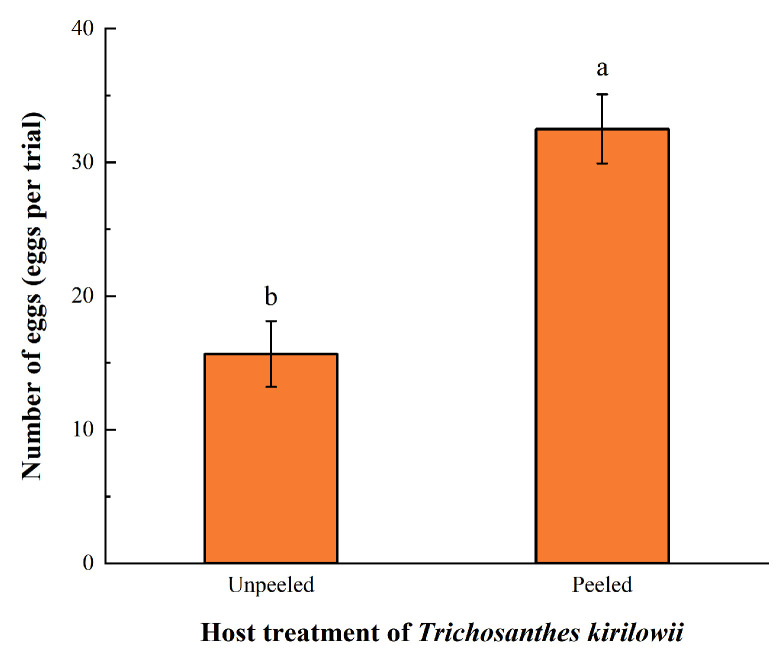
Fecundity of *Z. tau* on peeled and unpeeled *T. kirilowii*. Note: Different lowercase letters in the figure indicate significant differences between different treatments (*t*-test, *p* < 0.05). Values in the figure are expressed as mean ± standard error (mean ± SE).

Based on the oviposition data of *Z. tau* on the two host plants under both unpeeled and peeled conditions (Table 2), the number of eggs laid on *T. kirilowii* was significantly higher than that on *C. moschata* (both *p* < 0.001). Moreover, in the effect-comparison group (Figure 1), the mean number of eggs laid per trial on peeled *T. kirilowii* reached 32.50 ± 2.59 eggs per trial, which was significantly higher than that on unpeeled *T. kirilowii*.

### 3.3. Biological Parameters of Z. tau on T. kirilowii and C. moschata

#### 3.3.1. Developmental Duration

The developmental durations of *Z. tau* on *T. kirilowii* and *C. moschata* are shown in Table 3. No significant differences were detected in the duration of each developmental stage or total longevity between individuals reared on the two hosts (*p* > 0.05).

#### 3.3.2. Survival Rate at the Pre-Adult Stage

**Figure 2:**
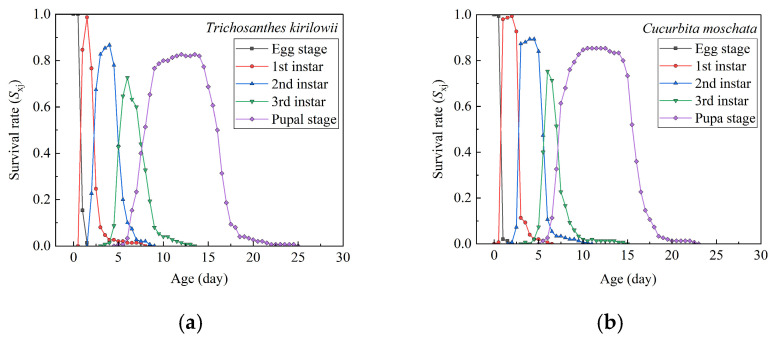
Age–stage-specific survival rates (*S_xj_*) of *Z. tau* on *T. kirilowii* (**a**) and *C. moschata* (**b**).

As shown in Figure 2, the *S_xj_* values of *Z. tau* on *T. kirilowii* and *C. moschata* varied between developmental stages, reflecting individual differences in developmental rates. On *C. moschata*, larval development was complete at approximately 14.5 d, with adult emergence occurring around 22.5 d, whereas larval development was complete at approximately 13.0 d on *T. kirilowii*, with adult emergence occurring around 24.5 d. The overlap of the *S_xj_* curves indicates variability in developmental timing among individuals within the population across different stages.

#### 3.3.3. Survival Rate at the Adult Stage

**Figure 3:**
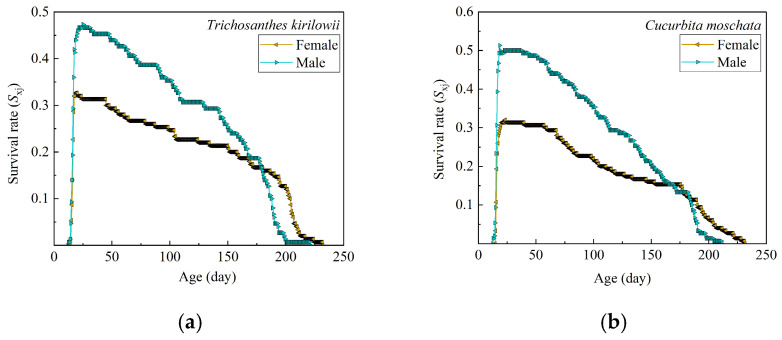
Age–stage-specific survival rates (*S_xj_*) of female and male *Z. tau* adults on *T. kirilowii* (**a**) and *C. moschata* (**b**).

In both hosts, the *S_xj_* values of female and male *Z. tau* adults peaked soon after emergence and then showed a declining trend. Within the same host, clear differences in *S_xj_* values were observed between females and males, as shown in Figure 3. On *T. kirilowii*, female survival declined to zero at 232 days, while males survived up to 221 days. Similarly, on *C. moschata*, female survival reached zero at 231.5 days, while males survived up to 211.5 days.

#### 3.3.4. Reproductive Parameters and Pupal Weight

As shown in Table 4, no significant differences were observed in the actual oviposition periods of *Z. tau* adults reared on *T. kirilowii* and *C. moschata*. In contrast, the APOP and TPOP were significantly shorter in the *T. kirilowii* group than in the *C. moschata* group (both *p* = 0.001). No significant differences were detected between the two host plants in terms of OP and hatching rate. By contrast, fecundity was significantly higher in the *T. kirilowii* group compared to the *C. moschata* group (*p* = 0.001), and pupal weight was also significantly greater in individuals reared on *T. kirilowii* (*p* = 0.001).

#### 3.3.5. Age-Specific Survival Rate and Fecundity

**Figure 4:**
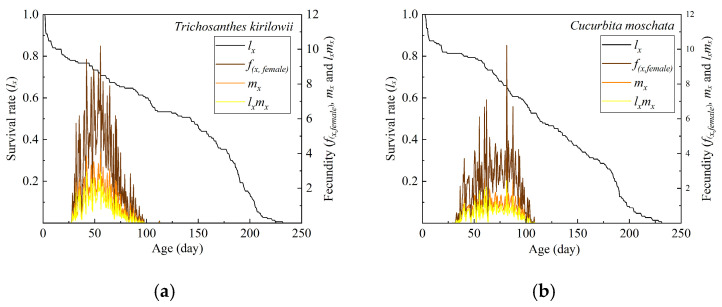
Reproductive and survival rates of *Z. tau* on *T. kirilowii* (**a**) and *C. moschata* (**b**).

The effects of the two hosts on the survival and fecundity parameters (*l_x_*, *f_x_*_,_*_f__e__male_*, *m_x_*, and *l_x_m_x_*) of *Z. tau* are presented in Figure 4. On both *T. kirilowii* and *C. moschata*, *l_x_* declined with age, and the last adult died at 231.5 d; however, the decline was slower on *T. kirilowii* than on *C. moschata*. On *T. kirilowii*, *f_x_*_,_*_female_*, *m_x_*, and *l_x_m_x_* all peaked at 55.5 d, with corresponding values of 10.1860, 4.0935, and 2.92; on *C. moschata*, these parameters all peaked at 81.5 d, with corresponding values of 10.2222, 3.7551, and 2.4533. Fluctuations in *m_x_* suggested that oviposition ceased at certain ages.

#### 3.3.6. Age–Stage-Specific Life Expectancy

**Figure 5:**
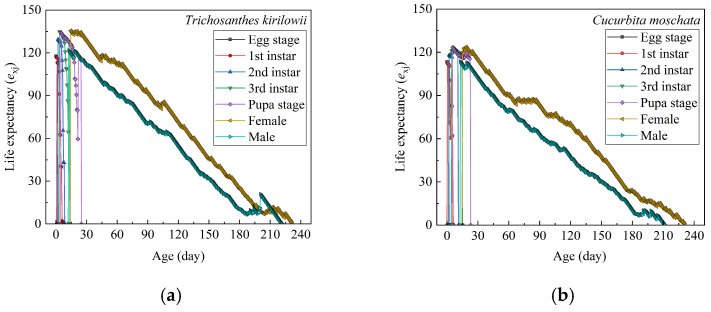
Age–stage-specific life expectancies (*e_xj_*) of *Z. tau* on *T. kirilowii* (**a**) and *C. moschata* (**b**).

The *e_xj_* values of *Z. tau* on both *T. kirilowii* and *C. moschata* show a declining trend with age (Figure 5). At the ages of 0, 1 days, the mean maximum life expectancy of *Z. tau* is 117.72 d on *T. kirilowii* and 113.27 d on *C. moschata*, which is consistent with the total longevity reported in Table 3.

#### 3.3.7. Age–Stage-Specific Reproductive Value

**Figure 6:**
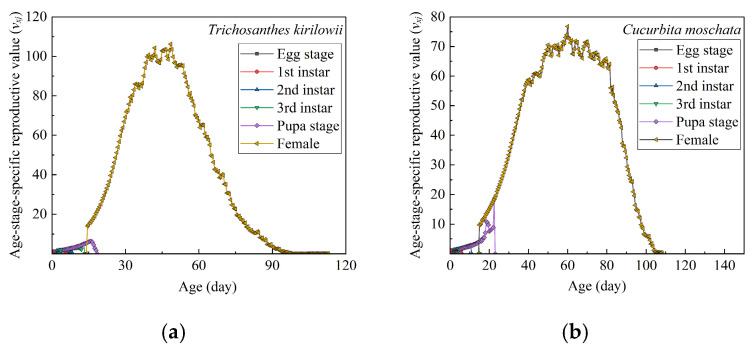
Age–stage-specific reproductive values (*v_xj_*) of *Z. tau* on *T. kirilowii* (**a**) and *C. moschata* (**b**).

The *v_xj_* values of *Z. tau* on both *T. kirilowii* and *C. moschata* are shown in Figure 6. At age 0, *v*_0,1_ was 1.05274 on *T. kirilowii* and 1.03926 on *C. moschata*. The *v_xj_* values showed that the peak reproductive value on *T. kirilowii* occurred at 48.5 d, whereas on *C. moschata* it occurred at 69.5 d.

#### 3.3.8. Key Population Parameters

Life table population parameters reflect the population growth and reproductive performance of *Z. tau* on *T. kirilowii* and *C. moschata*, as shown in Table 5. The *r* values of *Z. tau* were greater than zero and both the finite rate of increase *λ* and the net reproductive rate *R*_0_ were greater than one, indicating that the species was able to complete development and reproduction on both hosts. On *T. kirilowii*, the values of *r* and *λ* were significantly higher than those on *C. moschata* (*p* < 0.001). In contrast, no significant difference in *R*_0_ was observed between the two hosts (*p* > 0.05). In addition, the mean generation time of *Z. tau* on *C. moschata* was significantly longer than that on *T. kirilowii* (*p* < 0.001).

### 3.4. Population Size Prediction

**Figure 7:**
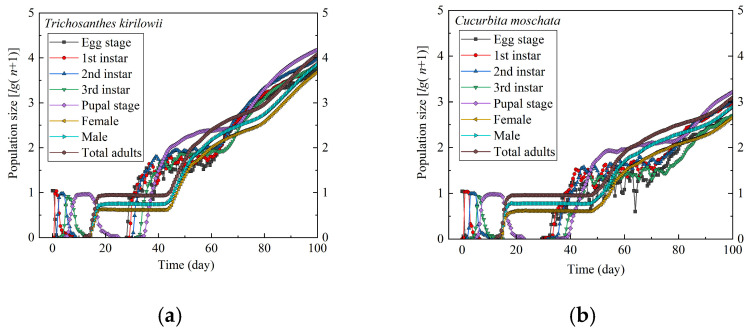
Predictions of population growth and structure of *Z. tau* on *T. kirilowii* (**a**) and *C. moschata* (**b**).

The population dynamics of *Z. tau* on different host plants over the next 100 d were predicted using the TIMING-MSChart (Ver. 2/5/2025) software, as shown in Figure 7. In the projections, adults first appeared at approximately 12.5 d on *T. kirilowii* and at 13 d on *C. moschata*. Overall, the predicted population size increased over time on both host plants, with different growth patterns observed between hosts. After 100 d of oviposition, the projected number of adults on *T. kirilowii* reached 11,980, whereas only 1231 adults were projected on *C. moschata*.

## 4. Discussion

The relationship between insects and plants has long been a key topic in insect ecology, with host selection widely regarded as an outcome of their long-term interaction and coevolution. Feeding and host finding are key behavioral processes that strongly influence an insect’s colonization success and reproductive performance throughout its life history [40]. Previous studies have shown that different tephritid fruit flies often exhibit pronounced host preferences under multi-host conditions. For instance, Amin et al. reported that *B. dorsalis* showed the highest preference for *Prunus persica*, followed by *Mangifera indica*, and the lowest preference for *Momordica charantia* [41]. On the other hand, Shahzadi et al. demonstrated significant differences in host selection by *Bactrocera cucurbitae* among cucurbit host species [42].

Our results indicate that *Z. tau* exhibited the strongest preference for and highest oviposition output on *T. kirilowii*, which also significantly increased its population growth potential. Differences in host suitability are commonly linked to host nutritional quality, profiles of volatile compounds, and long-term insect–host adaptation [43,44]. For example, Roy reported that feeding on a high-quality host can markedly accelerate population growth; *Bactrocera dorsalis* (Hendel) individuals developed most rapidly and exhibited the highest survival rate, fecundity, and population growth rate on guava among four tested hosts [45]. Accordingly, nutritional factors such as sugars and available carbon sources often play a critical role in accelerating development and enhancing fecundity. As a medicinal and edible plant, *T. kirilowii* possesses a relatively rich nutritional profile: Zhang et al. quantified major carbohydrates in its various tissues using UHPLC–MS/MS and reported high proportions of glucose (22.91%), fructose (20.63%), and polysaccharides (27.29%) in the fruit pulp [46]. This carbohydrate-rich composition likely provides a stronger energy base for larval growth, thereby improving developmental performance and reproductive output on *T. kirilowii*. In contrast, nutritional studies indicate that total and soluble sugar contents in *C. moschata* pulp are relatively low, approximately 1–5% on a fresh-weight basis or 10–70 mg/g on a dry-weight basis [47].

These comparisons suggest that polysaccharides and related compounds in *T. kirilowii* may offer a more sufficient energy supply for larval development. Correspondingly, differences in pupal weight, female fecundity, and oviposition timing were observed between hosts, with pupae being heavier, fecundity higher, and the oviposition peak occurring earlier on *T. kirilowii.* These patterns may indicate a potential association between nutritional compatibility and both individual performance and population-level reproduction. Beyond nutrients, chemical signals may further amplify host advantages by influencing adult orientation and oviposition site selection. In our odor-choice assays, adults showed the strongest attraction to peeled *T. kirilowii*. Xu et al. demonstrated that 1-octen-3-ol from mango volatiles can elicit female oviposition behavior in *B. dorsalis* and identified an olfactory receptor regulating this response [48]. Notably, previous studies on volatiles from *T. kirilowii* seeds have also reported that 1-octen-3-ol is a key component [49]. Thus, peeling *T. kirilowii* may enhance volatile emissions or alter its profile, increasing attractiveness to *B. tau* and improving the efficiency of oviposition site location, thereby promoting rapid colonization and reproductive potential on a suitable host.

Additionally, a co-adaptive background may exist between *Z. tau* and *T. kirilowii*. *Trichosanthes* plants have long been distributed across East and South Asia, providing a stable ecological context for sustained insect utilization [50]. From the perspective of crown-group age, *Trichosanthes* predates *Cucurbita*, suggesting that it represents an older Asian cucurbit lineage [51]. In comparison, *Cucurbita* crops originated in the Americas and were introduced relatively late into Asia [52]. Evidence also suggests that *Z. tau* (synonym: *Bactrocera tau*) has undergone a relatively long process of colonization and dispersal in Southern China, which has also been considered a possible part of its native distribution range [53]. Therefore, against the dual background of geographic co-occurrence and long-term utilization, *Z. tau* may have developed a higher level of physiological adaptation to *T. kirilowii*, as reflected in the shortened generation time observed in this study. Similarly, Wang et al. reported that fruit flies tend to colonize plant species that are closely related to their ancestral hosts [54]. Differences in larval survival and population growth were also observed, which may further indicate a potential association with host adaptation. These findings suggest that the *Z. tau* preference for *T. kirilowii* is not accidental but reflects its long-term adaptation to cucurbit hosts in Southern China and also provides strong support for the hypothesis that *Z. tau* is not an introduced invasive pest in China.

However, host-feeding preference and oviposition choice alone are insufficient to assess long-term outbreak risk [55]. Therefore, we applied the age–stage, two-sex life table theory and the TWOSEX-MSChart program to comprehensively quantify survival, development, and reproduction under different host conditions. Life table comparisons revealed that, although no significant difference in oviposition duration was observed between hosts, *Z. tau* reared on *T. kirilowii* exhibited significantly shorter APOP and TPOP, indicating that individuals entered the reproductive phase earlier. Similar patterns have been reported in other insect–host systems. Anamika Koner et al. found that *Galerucella placida* reared on *Rumex dentatus* exhibited shorter APOP and TPOP along with higher fecundity [56], suggesting that host plant nutritional quality can influence population performance by regulating reproductive timing. Furthermore, on *T. kirilowii*, the peak of the age–stage-specific reproductive value (*v_xj_*) of *Z. tau* occurred earlier, indicating that individuals reached their maximum contribution to population growth at an earlier age on this host. This result provides temporal evidence that earlier reproductive onset shifts the contribution to population growth forward. Differences in individual reproductive traits among hosts can accumulate at the population level, thereby influencing overall population growth. Consistent with this, *Z. tau* reared on *T. kirilowii* exhibited significantly higher intrinsic (*r*) and finite rates of increase (*λ*), along with a shorter mean generation time (*T*), indicating faster generational turnover and greater population expansion potential on *T. kirilowii* [2].

This pattern aligns with the strong preference for *T. kirilowii* observed in oviposition choice assays, suggesting that host preference may extend beyond short-term behavioral responses and contribute to population growth through enhanced reproductive output and faster generational turnover. The odor-choice assays further showed that the pronounced attraction to *T. kirilowii* could facilitate host location and potentially shorten the pre-oviposition period, thereby further supporting population establishment. Population projections based on the age–stage, two-sex life table provide insight into stage-structured dynamics and offer time-window information relevant to pest risk assessment and integrated management [25,37,57]. These projections indicated a faster population increase on *T. kirilowii*, accompanied by a rapid accumulation of individuals over time, suggesting a potential outbreak risk. In addition, temporal patterns of larval peaks and adult emergence were identified, which were consistent with developmental trajectories and the *S_xj_* curves. This, in turn, offers a critical indication for controlling timing within integrated pest management (IPM) programs [16].

## 5. Conclusions

*Z. tau* females showed a significant preference for *T. kirilowii*, whereas males showed no significant preferences. Oviposition was also higher on *T. kirilowii*. The developmental duration, total longevity, ovipositing days, oviposition period, and hatching rate did not differ between the two host species. However, on *T. kirilowii*, the APOP and TPOP were significantly shorter, and the average pupal weight was greater. In addition, we observed higher intrinsic and finite rates of increase and a shortened mean generation time but a similar net reproductive rate (*R*_0_). Taken together, these results indicate that *T. kirilowii* is a favorable host for *Z. tau*, with its advantages primarily manifesting through population performance parameters.

## Figures and Tables

**Table 1:** Number of female and male *Z. tau* adults on different cucurbit hosts under unpeeled and peeled treatments.

Treatment	Unpeeled		Peeled	
	Female	Male	Female	Male
*T. kirilowii.*	1.20 ± 0.47 a	1.10 ± 0.23 a	3.60 ± 0.65 a	1.70 ± 0.34 a
*C. moschata*	0.20 ± 0.13 b	1.50 ± 0.27 a	1.90 ± 0.31 b	1.00 ± 0.26 a

Note: Different lowercase letters in the same column indicate significant differences between different hosts (*t*-test, *p* < 0.05). Values in the table are expressed as mean ± standard error (mean ± SE).

**Table 2:** Oviposition preferences of *Z. tau* adults on *T. kirilowii* and *C. moschata*.

Treatment	Unpeeled	Peeled
*T. kirilowii*	22.17 ± 3.07 a	39.83 ± 4.59 a
*C. moschata*	0.33 ± 0.21 b	3.33 ± 0.49 b

Note: Different lowercase letters in the same column indicate significant differences (*t*-test, *p* < 0.05). Values in the table are expressed as mean ± standard error (mean ± SE).

**Table 3:** Developmental duration of *Z. tau* on *T. kirilowii* and *C. moschata*.

Developmental Stage	*n*	*T. kirilowii* (Days)	*n*	*C. moschata* (Days)
Egg	150	1.08 ± 0.02 a	150	1.01 ± 0.01 a
1st instar	134	1.48 ± 0.05 a	141	2.04 ± 0.03 a
2nd instar	127	2.87 ± 0.05 a	135	2.83 ± 0.05 a
3rd instar	126	2.57 ± 0.10 a	131	1.81 ± 0.07 a
Pupa stage	124	8.59 ± 0.06 a	129	8.60 ± 0.05 a
Pre-adult stage	124	16.52 ± 0.13 a	129	16.29 ± 0.12 a
Total longevity	124	117.72 ± 6.18 a	129	113.27 ± 5.63 a

Note: Same lowercase letters in the same row indicate no significant differences at the 0.05 level, as determined by the bootstrap procedure (*B* = 100,000). Values in the table are expressed as mean ± standard error (mean ± SE).

**Table 4:** Reproductive parameters and pupal weight of *Z. tau* on *T. kirilowii* and *C. moschata*.

Host	Ovipositing Days	APOP (Days)	TPOP (Days)	OP (Days)	Hatching Rate (%)	F (*n*/Individual)	P Weight (mg/Individual)
*T. kirilowii*	8.28 ± 0.41 a	19.46 ± 0.97 b	35.81 ± 0.99 b	43.31 ± 3.58 a	68.21 ± 0.23 a	381.24 ± 25.78 a	18.19 ± 0.53 a
*C. moschata*	8.41 ± 0.59 a	24.23 ± 0.91 a	40.72 ± 0.94 a	47.24 ± 3.54 a	70.46 ± 0.33 a	276.58 ± 23.31 b	12.91 ± 0.53 b

Note: Different lowercase letters in the same column indicate significant differences at the 0.05 level. Pupal weight and hatching rate were determined using a *t*-test, while the remaining parameters were analyzed using the bootstrap procedure (*B* = 100,000). Values in the table are expressed as mean ± standard error (mean ± SE).

**Table 5:** Population parameters of *Z. tau* on *T. kirilowii* and *C. moschata*.

Host	Intrinsic Rate of Increase *r* (d^−1^)	Finite Rate of Increase λ (d^−1^)	Net Reproductive Rate *R*_0_	Mean Generation Time *T* (d)
*T. kirilowii*	0.1028 ± 0.0040 a	1.1083 ± 0.0044 a	127.08 ± 16.97 a	47.13 ± 0.97 b
*C. moschata*	0.0770 ± 0.0032 b	1.0801 ± 0.0035 b	92.19 ± 13.18 a	58.74 ± 1.26 a

Note: Different lowercase letters in the same column indicate significant differences at the 0.05 level according to the bootstrap test (*B* = 100,000). Values in the table are expressed as mean ± standard error (mean ± SE).

## Data Availability

The original contributions presented in this study are included in the article. Further inquiries can be directed to the corresponding author.
